# Obituary

**Published:** 1909-11-06

**Authors:** 


### Obituary.

We have to record the death, at the age of 45, of Dr. Charles Reid. He studied at Edinburgh, and took his M.B. and C.M. in 1888. He had been in practice at Swindon for six years, and was well known in the sporting world as having been captain of the Scottish Rugby team for several years.

The death is announced of Mr. Percy A. H. Radcliffe, by drowning in the Epuhrates. Since 1907 he had been attached to the Mission of the C.M.S. at Bagdad. He took his M.B. at Leeds in 1901, and the Diploma of Tropical Medicine at Liverpool in 1905. He was once, also, ophthalmic house surgeon at the General Infirmary, Leeds, and house surgeon at the Birmingham Eye Hospital.

We have to record the death on October 2, at Mafeking, South Africa, of Mr. William Andrew Hayes, M.R.C.S., L.R.C.P., aged forty-eight. He studied at University College and took the conjoint diploma in 1892. Three years later he went out to South Africa, where he practised at Mafeking till 1905. Till his death he was engaged in post-graduate work on the opsonic methods.

